# Problems encountered when defining Arctic amplification as a ratio

**DOI:** 10.1038/srep30469

**Published:** 2016-07-27

**Authors:** Alistair Hind, Qiong Zhang, Gudrun Brattström

**Affiliations:** 1 Department of Mathematics, Stockholm University, Stockholm, 10691, Sweden; 2 Department of Physical Geography, Stockholm University, Stockholm, 10691, Sweden; 3 Bolin Centre for Climate Research, Stockholm University, Stockholm, 10691, Sweden

## Abstract

In climate change science the term ‘Arctic amplification’ has become synonymous with an estimation of the ratio of a change in Arctic temperatures compared with a broader reference change under the same period, usually in global temperatures. Here, it is shown that this definition of Arctic amplification comes with a suite of difficulties related to the statistical properties of the ratio estimator itself. Most problematic is the complexity of categorizing uncertainty in Arctic amplification when the global, or reference, change in temperature is close to 0 over a period of interest, in which case it may be impossible to set bounds on this uncertainty. An important conceptual distinction is made between the ‘Ratio of Means’ and ‘Mean Ratio’ approaches to defining a ratio estimate of Arctic amplification, as they do not only possess different uncertainty properties regarding the amplification factor, but are also demonstrated to ask different scientific questions. Uncertainty in the estimated range of the Arctic amplification factor using the latest global climate models and climate forcing scenarios is expanded upon and shown to be greater than previously demonstrated for future climate projections, particularly using forcing scenarios with lower concentrations of greenhouse gases.

When the magnitude of zonally averaged surface temperature change at high latitudes exceeds globally averaged temperature changes in response to climate forcings on interannual or longer time scales, we often refer to this as polar amplification, of which Arctic amplification is the northern part. At least, this is the definition considered in the IPCC (Intergovernmental Panel on Climate Change) fifth assessment report (AR5)^1^, which broadly reflects the current standing of the scientific community on the state of climate research. Arctic amplification is evident in instrumental observations^2^, climate model simulated temperatures^3^,^4^ as well as historical occurrences based on palaeoclimatic evidence^5^,^6^. It is often claimed that the near surface of the Northern Hemisphere high latitudes are warming at rates roughly double those of the global average in recent decades, particularly in the autumn/winter^7^,^8^,^9^,^10^. Similar degrees of amplification also occur in climate model simulations of future projections forced with higher greenhouse gas concentrations^1^,^11^,^12^,^13^. These results however are frequently based on the analysis of different time-periods, different regional definitions and include different treatments of uncertainty. Motivation for this article comes in many respects from a lack of standardized results in Arctic amplification analysis, which can result in confusion regarding the uncertainty of the amplification factor.

The myriad of potential and known causes, feedbacks or drivers involved in this amplification process are not the topic of interest in this article. For a comprehensive overview of that topic we refer the reader to Serreze & Barry^14^, as well as Lu & Cai^15^ and Pithan & Mauritsen^4^. Neither will we presently cover issues pertaining to palaeoclimatic reconstructions of Arctic amplification. Here the problem of defining Arctic amplification itself is dealt with, attempting a synthesis of present definitions discussing the pitfalls and benefits with these approaches. Various aspects of complexity in the distribution of the ratio estimator statistic are discussed, in the context of current research focusing on Arctic amplification. Rather than being just a case of statistical detail, we stress that understanding of the behavior of the uncertainty in the ratio estimator involves fundamental concepts regarding the scientific questions being addressed. An approach to develop confidence intervals for ratio estimators is demonstrated, which as an example based on results from IPCC AR5, gives a clearer picture of how uncertainty in Arctic amplification in future projections from climate models is related to the degree of external radiative forcing. The range of definitions used to quantify the Arctic amplification factor are reviewed, as well as comparatively underused alternative definitions to the ratio estimator when considering the concept of amplification of temperatures at higher latitudes.

## Ratio Estimators

The ratio estimator approach to defining Arctic amplification is most often expressed as the mean change in Arctic temperatures (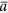) divided by the global change (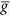) over a period of interest, so that an estimate of Arctic amplification (*r*), capable of being either positive (a change to warmer climate) or negative (a change to colder climate), can be represented as:


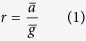


Note that global and Arctic changes in temperature 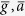 can be considered over any length of time, using any time resolution, however it is often multi-decadal trends or changes, based on seasonally or annually averaged data that are considered in the examples given in this article. Despite that eq. (1) is often used to define Arctic amplification, many may not be aware that the probability distribution of this ratio estimator can exhibit a complicated behavior, even if uncertainties in 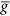 and 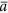 are well quantified^16^,^17^,^18^. This complicated probability distribution for ratio estimators can occur if the denominator in a given ratio estimator, for the purposes of this article–global temperature change (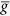), is close to 0 relative to its distribution. This makes estimation of uncertainty bounds (or confidence intervals) for ratio estimators problematic. The Fieller method or “Fieller’s theorem” is a practiced solution known in statistics for specifying confidence intervals for ratios and can be used to illustrate this complicated behavior^19^. Note that there are other possible approaches to confidence intervals for ratios, however provided the means of the numerator and denominator are approximately normally distributed, Fieller’s theorem is a practical solution, even for smaller sample sizes^20^.

The derivation of confidence intervals for ratios using Fieller’s theorem is described more thoroughly in the ‘Methods detail’ section at the end of this article, where most importantly we see that “bounded” confidence intervals can only be obtained when the denominator tends to be significantly different from 0^21^. Where the denominator is likely to *not* be significantly different from 0, then the absolute value of the ratio estimator may be arbitrarily inflated or even change signs. Note that if the numerator, in this case Arctic temperature change (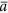), is not significantly different from 0 either, then essentially *any* value of the Arctic amplification factor is possible.

## Ratio of Means or Mean Ratio

The ratio estimator presented so far is also known as the ‘Ratio of Means’ estimator (*r*_R_), which functions literally as the ratio of two estimated means:


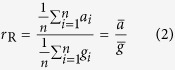


The sample mean global (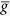) and Arctic (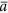) temperature changes can be estimated from climate models *i* (in an ensemble of size *n*) for example. Contrast this with the ‘Mean Ratio’ estimator (*r*_M_), where an estimation of mean Arctic amplification is made based on the ratio estimate of each individual model simulation *i*:


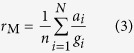


**Figure 1:**
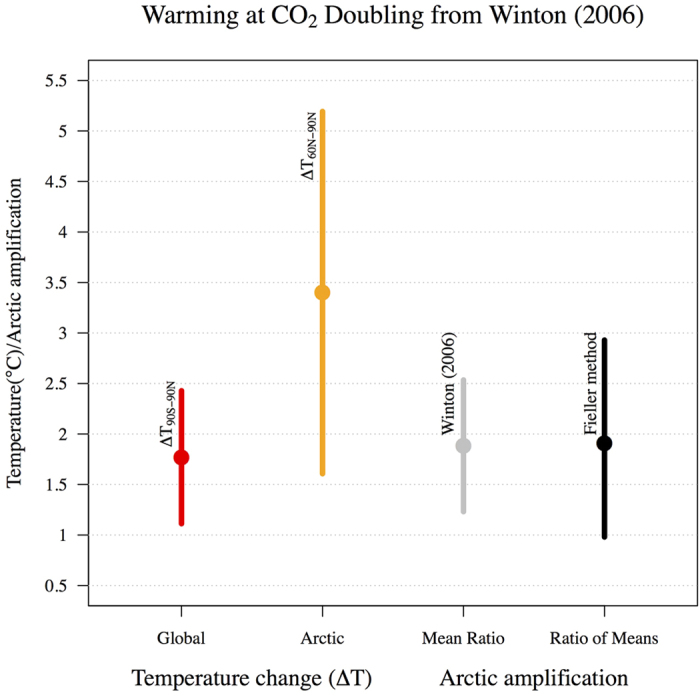
Based on Winton’s^12^ (WN) Fig. 1, where mean global and Arctic temperature change as well as Arctic amplification factors are presented for a doubling of carbon dioxide (CO_2_) equilibrium experiment based on twelve climate model simulations from CMIP4 (first three columns). Note that the original figure presented respective standard deviations, whereas here we present the 90% confidence interval ranges. The fourth column of data is a Ratio of Means definition of Arctic amplification with model spread calculated using the methodology presented in Fieller^19^ as opposed to the Mean Ratio approach employed by the original authors (column 3).

At first glance, the differences between eqs (2) and (3) may appear trivial, however these estimators ask different scientific questions when estimates are based on climate model ensembles (or alternative observational datasets). We use Winton^12^ (abbreviated as WN) as an example of the ‘Mean Ratio’ estimator approach to Arctic amplification though there are other examples in the literature^11^,^22^. In WN twelve model simulations taken from the CMIP4 (Coupled Model Intercomparison Project) ensemble were used to estimate an Arctic amplification factor, where the models were allowed to reach a quasi-equilibrium state after being subjected to a doubling of carbon dioxide (CO_2_) (Fig. 1). They first calculated the Arctic amplification of each of their individual twelve (*n* = 12) ensemble members 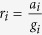, then formed sample mean ratio and uncertainty statistics of the distribution based on the model spread of these ratios (eq. (3), column 3 in Fig. 1). This particular example in WN corresponds to an investigation of the average Arctic amplification factor in a climate model ensemble after responding to a simulated doubling of CO_2_. If however a ‘Ratio of Means’ estimator (eq. (2)) approach is used in WN, the investigation becomes quite conceptually different in regards to both the amplification factor and the uncertainty therewith. Using a ‘Ratio of Means’ approach would rather mean investigating how the average Arctic temperature change response to a simulated doubling of CO_2_ is comparable with the global average. In other words, the estimated uncertainty in the Arctic amplification factor using a ‘Mean Ratio’ approach is based on the spread of individual climate model or data-set Arctic amplification factors (*r*_*i*_), whereas the estimated uncertainty for the ‘Ratio of Means’ approach compares the spread of Arctic temperature changes (*a*_*i*_) from different climate models or data-sets with the respective spread of global changes (*g*_*i*_). Hence, Fieller’s theorem for deriving confidence intervals for ratios corresponds to the Ratio of Means estimator exclusively. The difference between the ‘Mean Ratio’ and ‘Ratio of Means’ approaches applied to WN’s model ensemble are illustrated in Fig. 1 (column 3 against column 4), where it can clearly be seen that the uncertainty increases using the latter approach.

If an average ratio estimate of Arctic amplification is sought, both the Mean Ratio and Ratio of Means will likely provide similar results (*r*_M_ ~ *r*_R_), provided denominator values close to 0 are considered extremely unlikely. For example, compare the similar mean Arctic amplification factors calculated using both approaches in columns 3 and 4 of Fig. 1 (Mean Ratio and Ratio of Means respectively). If however global temperature changes are likely close to 0 relative to the distribution of 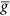, then *r*_M_ can be sensitive to outlying values in any individual model Arctic amplification factor (*r*_*i*_) due to the fact that the Mean Ratio estimator (*r*_M_) gives equal weighting to individual ratio estimates in the ensemble. Consider expressing eq. (1) in the form 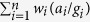, in which case 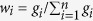 for the Ratio of Means (*r*_R_) and when *w* = 1/*n* we have the Mean Ratio (*r*_M_). It is clear the latter ratio estimator gives equal weighting to each individual ratio 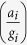, even if individual ratios may be highly inflated (see Supplementary Information). Furthermore, Rao *et al.*^23^ demonstrated through a second-order Taylor expansion of the ratio estimator that as sample size *n* increases, the expected value of the Ratio of Means (*E*[*r*_R_]) approaches 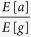, whereas the expected Mean Ratio statistic (*E*[*r*_M_]) does not. As a result the Mean Ratio (*r*_M_) can be considered an inconsistent estimator of 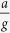
24 (demonstrated in Supplementary Information).

There are certainly conceptual differences between the Ratio of Means (*r*_R_) and Mean Ratio (*r*_M_) estimators of Arctic amplification. As demonstrated using WN’s data in the above example, the Ratio of Means gives larger confidence intervals than the Mean Ratio (Fig. 1) as it encompasses intermodel (from a multi-model ensemble encompassing several different physical climate models) uncertainty in both the Arctic and global temperature change estimates, instead of intermodel uncertainty in the Arctic amplification factor itself. Each individual multi-model ensemble member has a particular climate sensitivity to a given radiative forcing^25^, which means that resulting Arctic and global temperature changes can be compared for different individual model climate sensitivities within the multi-model ensemble when using the Ratio of Means approach. It is important to understand that here we are discussing uncertainty in the spread of a climate model ensemble, not the *statistical* uncertainty, or precision, of the ratio estimators themselves. The statistical uncertainty relates to how precisely a given sample ratio estimator 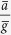 represents a true population ratio 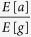, in which case the Ratio of Means is actually less *statistically* uncertain than the Mean Ratio^26^.

If a given analysis wishes to investigate how the projected temperature change in the Arctic compares with global changes, then confidence intervals for the Ratio of Means seems appropriate, as you would be most interested in comparing estimates of changes in both regions, even if it is perhaps the case that no individual member of the model or data-set ensemble is capable of producing results at the tails of the distribution. The Mean Ratio approach on the other hand, only allows Arctic and global regions to be compared for a given individual model or data-set, with the same climate sensitivity. Given the conceptual differences in these approaches, deciding which is more appropriate to use is rather dependent on the nature of the specific analysis question. The Mean Ratio certainly seems appropriate when comparing the individual Arctic amplification factors of members in a climate model ensemble or in different data-sets, however the aforementioned statistical inconsistency and susceptibility to outliers can be problematic, unless it is known that the denominator is unlikely to be close to 0 within its distribution.

## Confidence Intervals for Arctic Amplification in the IPCC Fifth Assessment Report (AR5)

**Figure 2:**
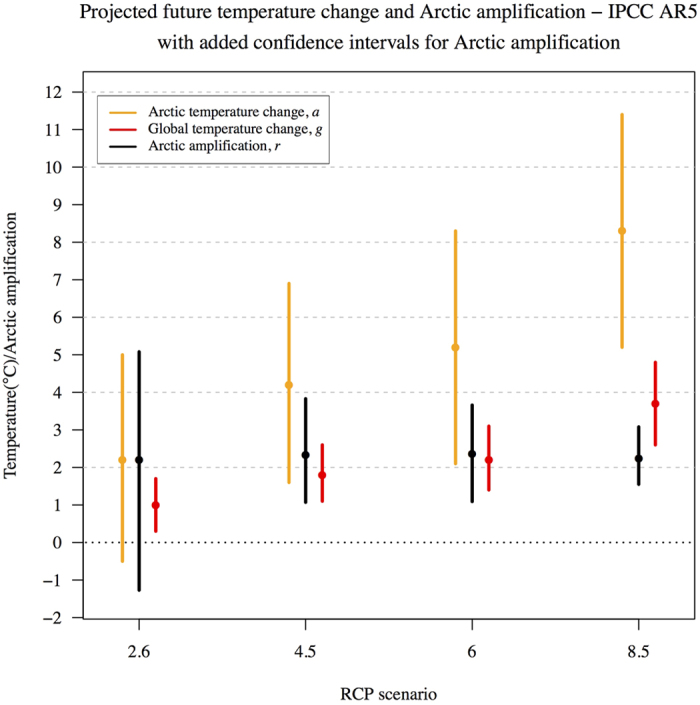
Projected simulated future temperature change between 2081–2100 and 1986–2005 from IPCC AR5 based on the respective Representation Concentration Pathways (RCPs, which are projected future forcing scenarios). The global (red) and Arctic (orange) mean change and intervals at the 90% confidence level (Gaussian assumption), as well as Ratio of Means Arctic amplification factors, are provided for each RCP forcing scenario based on the CMIP5 climate model ensemble data published in IPCC AR5. The authors provide the additional Arctic amplification (black) confidence intervals calculated using the Fieller method^19^, based on the CMIP5 model ensemble statistics. Note that the ensemble size differs for each forcing scenario (*n* = 32, 42, 25, 39 for RCP2.6, RCP4.5, RCP6, RCP8.5 respectively).

In IPCC AR5 projected future Arctic amplification based on the CMIP5 climate model ensemble is claimed to be “between 2.2–2.4 times the global average warming for 2081–2100 compared to 1986–2005” (see Table 12.2 in IPCC AR5^27^). This range of future projected Arctic amplification factors is based on the Ratio of Means estimate of four multi-model ensembles with different forcing scenarios known as Representative Concentration Pathways (RCPs), which are labeled as RCP2.6, RCP4.5, RCP6 and RCP8.5. The RCP numbers approximately refer to the maximum projected total global radiative forcing effect (*Wm*^2^) they are predicted to achieve before 2100, although RCP2.6 would likely peak around 3 *Wm*^2^ and significantly earlier than 2100^28^. Figure 2 shows the multi-model mean Arctic (orange/yellow) and global (red) temperature changes projected for the period 2081–2100 in contrast with those simulated for 1986–2005 using the four RCP scenarios. The 90% confidence intervals for the spread of Arctic and global temperatures are presented here as provided in Table 12.2 of IPCC AR5 and are essentially very similar for all RCP forcing scenarios. Here, we apply the Fieller method in an attempt to give representative confidence intervals at a 90% significance level for projected Arctic amplification using the Ratio of Means approach, based on the model spread for each RCP forcing scenario (black in Fig. 2). The RCP4.5 and RCP6 simulated future scenarios show a similar uncertainty in the Arctic amplification factor, with potential values ranging from approximately 1.2–3.8 at a 90% significance level (second and third columns in Fig. 2), whereas the RCP8.5 scenario has a somewhat smaller uncertainty, ranging from 1.5–3 (fourth column in Fig. 2). On the other hand, for the RCP2.6 simulated future scenarios an Arctic amplification factor of less than 1 or even negative values are quite possible. In other words, the Arctic region may be able to undergo temperature changes in opposition to the direction of any global changes if the global radiative forcing follows the RCP2.6 pathway. It generally seems as though the uncertainty bounds calculated for future projected Arctic amplification factors indicate that higher numbered RCP forcing experiments (analogous to higher greenhouse gas concentrations) show less uncertainty than the lower RCP experiments. This is perhaps not an unexpected result given that higher greenhouse gas forcings would be expected to increasingly overcome differences in the physical models and internal climate variability.

The potential for negative Arctic amplification factors in the spread of the RCP2.6 pathway simulations above is due to the fact that spread of the projected future global changes for this scenario ensemble is quite close to 0 at its lower end (first column in Fig. 2). Consider the hypothetical case of an additional projected radiative forcing scenario that would be weaker than RCP2.6, with mean temperature changes of 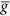 = 0.5 °C and 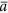 = 1.1 °C (compared to 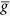 = 1.0 °C and 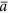 = 2.2 °C in the case of RCP2.6), and with the same model uncertainty in 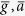 as RCP2.6 (a not unreasonable assumption given the similarity in model uncertainty for the four RCP forcing scenarios as seen in Fig. 2). For this hypothetical weaker RCP scenario it is impossible to generate bounded confidence intervals for Arctic amplification at all using the Fieller method, in which case the corresponding mean Arctic amplification factor (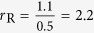) is rendered uninterpretable. This is simply because if the distribution of the denominator 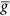 is close to 0, then artificially inflated values of Arctic amplification are possible that cannot be meaningfully interpreted^20^. The range of 2.2–2.4 presented in IPCC AR5 is a simple representation of four mean values based on the Ratio of Means estimator for each RCP forcing scenario and does not represent the full model uncertainty in future projected Arctic amplification; nor does it link uncertainty with different RCP radiative forcing scenarios as the confidence intervals calculated here for Arctic amplification clearly show in Fig. 2. Naturally, one must also consider the confidence level used to generate these statistics as increasing the confidence level will increase the chance of uninterpretable uncertainties in the ratio estimator, by increasing the width of the distribution of the denominator 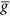. In the hypothetical case above, a number for an Arctic amplification factor can be generated from the mean temperature change statistics 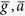 despite the result being essentially meaningless given the uncertainty.

## Defining the Arctic

There are a great deal of choices that can be made when investigating the nature of Arctic amplification in climate models or gridded observational datasets. A sample of studies from the literature that more or less focus on defining Arctic amplification are presented in Table 1. Note that we do not include investigations considering palaeodata given the markedly reduced data coverage. IPCC AR5 defines the Arctic as 67.5–90°N^27^ for example, in contrast with a slightly different definition in IPCC AR4^29^ of 65–90°N. There is also the issue of whether annual or seasonal data is used, given that Arctic amplification tends to be stronger during the autumn/winter months^8^,^9^,^30^. The reference region used in these analyses are included as well, namely if global or Northern Hemisphere temperature changes were used.

Spatial resolution of gridded data, as well as climate model specific factors, may influence the choice of region used to represent Arctic temperatures in order to generate Arctic amplification statistics. It is however rare in the literature that any justification of these choices are stated when regional averaging occurs. An investigation could certainly be undertaken to see how sensitive the Arctic amplification factor is to particular averaging choices made. There are cases in the literature where reference is made to more emphatic changing temperatures at higher latitudes as meaning Arctic amplification, but it is left to the reader to interpret what constitutes high latitudes (see Serreze & Francis^31^ or Yamanouchi^32^ for example). Consider one of the earliest multi-model ensemble estimated ranges given for Arctic amplification in Holland & Bitz^11^ (abbreviated as HB). A specific range of simulated polar warming for the Arctic region was claimed to be 1.5–4.5 times the global mean warming for a doubling of carbon dioxide (2 × CO_2_) in HB; and has been used as a benchmark for comparison with other Arctic amplification studies (s. 904 in IPCC AR4^33^). Hazeleger *et al.*^34^ used this 1.5–4.5 range to corroborate their simulated Arctic amplification factor of 2.5, however this was for an Arctic averaged over 70–90°N, whereas the stated HB range was actually the maximum and minimum calculated from 5° zonal bands. The 1.5–4.5 range quoted from HB is naturally larger than one would expect for amplification factors based on averaging over the entire Arctic region. A simple area-weighted averaging using HB’s data indicates rather a range of 1.8–2.7 for the Arctic region defined for 70–90°N, as an example of how Hazeleger *et al.*^34^ could have more fairly compared their results with the earlier example in HB.

## Discussion

An important distinction exists between the various analyses of Arctic amplification made so far; and that is whether they are considering changes in temperature as an equilibrium or transient climate response. Looking at annual to decadal temperature changes in observational data, or climate model simulations where radiative forcings are time dependent, is necessarily the reflection of a transient climate response, whereas an equilibrium climate response would require longer time scales so that the deep oceans have time to equilibriate with a given radiative forcing^35^. For example, the future projected temperature changes from IPCC AR5 are transient responses to the RCP radiative forcing scenarios, whereas the simulated doubling of CO_2_ concentrations in WN^12^ represent an equilibrium response, given that they wait for the climate model system to more or less equilibriate with a prescribed change in radiative forcing. Climate models that are run to some form of quasi-equilibrium, inherently tend to display an Arctic amplification (or more generally polar amplification)^36^. In contrast, transient simulations or indeed the observed climate system, may very well undergo periods where the global change is close to 0 or where there is no Arctic amplification at all and the global changes exceed changes in the Arctic (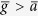). For example, Polyakov^37^ found observational evidence for a cooling Arctic during periods of warming in the Northern Hemisphere at large during the 20^th^ century whereas Chylek *et al.*^38^ found inflated Arctic amplification factors based on meteorological station data for the period 1940–1970, largely because the global temperature change for that period was very close to 0. Indeed, the concept of an amplification factor defined as a ratio can be questioned for shorter term transient responses in the climate system.

Bekryaev *et al.*^2^ (abbreviated as BV) recognized some of the complexity of the ratio estimator approach to Arctic amplification (specifically, where the denominator approaches 0) and suggest that Arctic amplification should rather be defined by the ordinary least squares (OLS) linear regression of temperature in the Arctic *T*_*a*_(*t*) on to Northern Hemispheric temperature (labeled *T*_*g*_(*t*) here for simplicity) in the form *T*_*a*_(*t*) = *r*_C_*T*_*g*_(*t*) + *b*, where *r*_C_ represents the Arctic amplification factor. Based on 42 historical runs from the CMIP3 model ensemble, BV found this linear regression approach to Arctic amplification to be stable over the model spread when compared with ratio estimation. A linear relationship between the Arctic and global temperatures certainly seems to exist based on equilibrium projection results of climate model ensembles^39^, but it is not clear whether this linearity can be assumed for a transient response of the climate system on shorter timescales (such as decadal) as well. Sensitivity to the choice of time period could also be investigated. An important issue with OLS regression of Arctic (dependent variable) on to global or hemispheric temperatures (independent variable) is that uncertainty is only assumed in Arctic temperatures, whereas uncertainty exists in both *a* and *g* (i.e. an errors-in-variables problem^40^). It seems reasonable to assume that uncertainty in hemispheric or global temperatures is at least within an order of magnitude of Arctic uncertainty^12^. We would suggest treating the covariates and independent variable in a more geometrically symmetrical manner, such as in total least squares (TLS) regression^41^, or a weighted TLS^42^ when it is known that the uncertainties are likely of a different size. Note that one could just as easily consider global temperatures as a linear function of Arctic temperatures instead, then the estimated regression coefficient (*r*_c_) would be larger than using the BV approach; and different errors-in-variables models like TLS or weighted TLS would be somewhere in between.

An early framing of Arctic amplification by Callendar^43^ was to compare higher and lower latitude bands, where the “temperature difference for 1921–1950 minus 1891–1920 for the latitude zone 60–73°N was 0.81 °C, compared with 0.39 °C for the band spanning 25–60°N”. However, consideration of Arctic amplification in terms of latitudinal differences in changes of temperature is uncommon in the literature, at least as a measured quantity. Notably, Crook *et al.*^44^ defined amplification as the difference in warming between polar and tropical regions for various physical contributions to polar amplification (such as radiative forcings and horizontal heat transports). Arctic or more generally, polar amplification exists where horizontal heat transport between polar and equatorial regions causes a latitudinal difference in temperature distribution that would not be predicted by local net radiation balances^45^. Considering this physical definition of amplification, a latitudinal difference in temperature changes perhaps seems more relevant to studies focusing on Arctic (or polar) amplification than using either hemispheric or global temperature changes as a reference. An issue would then be justification of what constitutes the Arctic or tropical regions of course. One way to distinguish the Arctic and tropical regions could be based on either side of where the (annually or seasonally) averaged meridional wind component *v* is equal to 0. This would at least allow the data-set or climate model to more decide where the higher and lower latitude regions lie.

## Summary

An attempt has been made to draw attention to many aspects that can potentially lead to confusion in defining Arctic amplification using a ratio estimator (*r*), and in particular, quantification of its uncertainty. When Arctic amplification is defined as a ratio estimator, it is usually the case that the numerator is representative of a change in temperatures over the Arctic (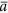) and the denominator is a reference change, such as in global temperatures (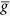). If the global temperature change is near to 0 the amplification factor can become artificially inflated and hence both statistically and physically meaningless; if Arctic temperature change is also near to 0 then essentially *any* value of Arctic amplification is possible.

Here, a distinction is made between the ‘Mean Ratio’ and ‘Ratio of Means’ estimators, which can also result in a misattribution of uncertainty in Arctic amplification. For a climate model ensemble, a sample uncertainty can easily be derived using the Mean Ratio estimator by looking at the model spread of the individual climate model’s Arctic amplification factors 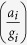, however the Mean Ratio estimator is statistically inconsistent, in that regardless of how large a model ensemble or set of alternative observational data are used, this statistic will not converge on the “expected” value of the Arctic amplification factor expressed as 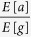, unlike the Ratio of Means estimator which does. Using a sample uncertainty based on the model spread of both Arctic and global temperature changes, confidence intervals can be derived for the Ratio of Means estimator using ‘Fieller’s theorem’^19^. The uncertainty derived from the Ratio of Means estimate of Arctic amplification in a multi-model ensemble tends to be larger than when using the Mean Ratio estimator, as demonstrated here on the climate model ensemble of Winton^12^. We speculate that the main reason for this increased uncertainty is that for the Ratio of Means estimator, changes in Arctic temperatures can be compared with global changes from two different climate models potentially possessing different climate sensitivities to radiative forcing. Therefore, if individual model Arctic amplification factors are calculated, as is done to define the Mean Ratio estimator, then uncertainty is conceptually limited by the range of individual model ensemble member climate sensitivities.

The IPCC fifth assessment report (AR5) presents the most up-to-date projections of future changes in Arctic temperatures to be some 2.2–2.4 those of projected global changes comparing the end of the 21^st^ century with the recent past^27^. We emphasize that this range of future temperature changes with respect to latitude presented in IPCC AR5 can be misleading. The IPCC AR5 report’s claimed 2.2–2.4 range of future projected Arctic amplification is actually just the range of the four *mean* amplification factors for four differently forced climate model ensembles (RCPs). These four mean values are calculated in IPCC AR5 using the Ratio of Means estimator. Here, it is demonstrated using Fieller’s theorem that the uncertainty in the Arctic amplification factor differs greatly between the four different RCP forcing experiments. In fact, the uncertainty in the amplification factor is more complicated than the 2.2–2.4 range presented in IPCC AR5 suggests, with lower greenhouse gas emitting future scenarios having greater uncertainty than the experiments with higher projected radiative forcing. In the case of RCP2.6, the future scenario with the lowest projected anthropogenic emissions and radiative forcing, the Arctic amplification factor could likely be less than 0 or even have a negative value due to the spread of future projected global temperature changes being close to 0 for this particular experiment, comparing the recent past with the end of the 21st century. This would understandably have both global and regional implications for mitigation and adaptation strategies under future climate change.

The fact that increased CO_2_ equilibrium temperature response experiments, recent future projected scenarios in IPCC AR5, as well as surface temperature observations over recent decades have all roughly shown a doubling of the rate of warming in the Arctic region compared with global temperature changes, has perhaps masked potential discussion regarding the ratio estimator as a suitable definition for the concept of amplification itself. The uncertainty in an Arctic (or polar) amplification factor expressed as a ratio is clearly far from trivial when changes in global temperature are near 0, such as can easily be observed in the climate system on inter-annual to decadal time-scales, or as can occur in transient climate model simulations.

## Methods Detail

### Fieller’s theorem

Consider Arctic and global temperature change where eq. (1) is expressed in the form 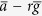. Following the derivation of Franz^21^, dividing 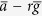 by an estimate of its standard deviation gives the following statistic:


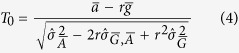


where 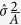, 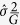 are the variance estimators for a change in Arctic and global temperatures respectively and 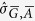 represents an estimate of their covariance. If both 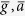 are assumed to be normally distributed, then so is the term 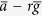, which consists of the difference of two normal variables. Note that normality is assumed for 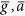 regardless of the number of observations (or model ensemble members). The *T*_0_ estimator therefore approximates a Student t-distribution, given that we are standardizing the mean of our normal data by a standard deviation which is itself estimated from the data. The distribution of *T*_0_ will resemble a normal distribution more and more as the number of observations increases. *T*_0_ follows a t-distribution exactly (again, assuming the data is normally distributed for 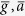) given that observations are of the form 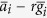 (as in Fieller’s theorem), which are independent for different *i* observations (or model ensemble members if you will) and require neither dependence or independence between the paired observations 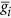 and 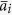 (paired because they come from the same climate model). These criteria are met in the case of the example model ensemble experiments used in this article.

To obtain approximate confidence intervals for *r*, one can calculate the set of *r* values for which the corresponding *T*_0_ values lie within the upper and lower (1 − *α*) quantiles of the t-distribution (these quantiles are denoted as *t*_*q*_ here and can be obtained from R function *qt* or Matlab function *nctinv*, where the *degrees of freedom* are specified as *n* − 2, *n* being the ensemble size). This can be expressed in terms of the inequalities −*t*_*q*_ ≤ *T*_0_ ≤ *t*_*q*_, the solving of which for *r* leads to the following confidence limits^20^:


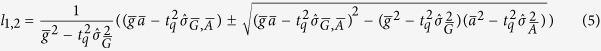


where *l*_1_ and *l*_2_ give “bounded” confidence intervals provided that the denominator, global temperature change (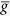), is different from 0 at significance level *α*. Otherwise a confidence set is obtained which only excludes the values between *l*_1_ and *l*_2_ (“unbounded/exclusive” case) or else a confidence set is obtained that does not exclude any values at all (“unbounded” case)^20^,^21^. Two quantities can be used to determine which class of confidence intervals within Fieller’s theorem are available for a given *r*, namely 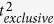 and 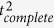, defined below as:


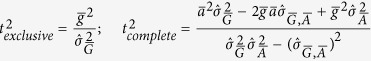


and which in the formulation of von Luxburg & Franz^20^ gives unbounded/exclusive confidence intervals if 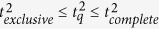 and unbounded confidence intervals where 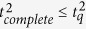, otherwise bounded confidence intervals can be determined. The Fieller’s theorem confidence interval sets can be summarized as:


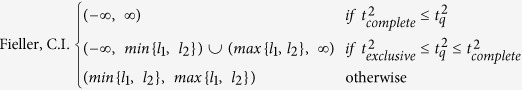


Note that the expression under the root sign in the numerator of eq. (5) will always be non-negative (as long as the correlation of errors in 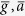 are not negatively correlated) whenever the denominator 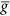 is significantly different from 0. 
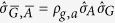
 from eq. (4) allows for correlation between the ratio denominator and estimator via *ρ*_*g*,*a*_. We used a correlation coefficient of *ρ*_*g*,*a*_ = 0.6 taken from the approximate correlation between the annual mean temperature series of the Arctic (60–90°N) and global regions based on HadCRUT4^46^,^47^ and GISTEMP^48^,^49^ data, to the nearest 0.05. Note that the dimensions of the confidence intervals were largely insensitive to the choice of *ρ*_*g*,*a*_ used, within the scope of the results given by the various observational datasets.

## Additional Information

**How to cite this article**: Hind, A. *et al.* Problems encountered when defining Arctic amplification as a ratio. *Sci. Rep.* **6**, 30469; doi: 10.1038/srep30469 (2016).

## Supplementary Material

Supplementary Information

## Figures and Tables

**Table 1:** A summary of key articles and reports relating to Arctic amplification and their respective areal definitions of the Arctic region; and whether the global or Northern Hemispheric (NH) temperature changes were used as a reference.

Definition of Arctic amplification			
Reference	Arctic region	Season	Relative to
IPCC (2007) AR4 [s. 249]^29^	>65°N	ann	global
IPCC (2013) AR5 [s. 1055]^27^	≥67.5°N	ann	global
Bekryaev *et al.* (2010)^2^	≥60°N	*all*	NH
Chylek *et al.* (2009)^38^	≥64°N	*all*	global
Crook *et al.* (2011)^44^ *	≥60°N	*all*	global
Graversen *et al.* (2008)^7^ **	>65°N	Nov–Feb	NH
Hazeleger *et al.* (2012)^34^	≥70°N	ann	global
Holland & Bitz (2003)^11^	≥75°N	ann	global
Hwang *et al.* (2011)^22^	>70°N	ann	global
Kelly (1982)^50^	65°N–85°N	ann	NH
Screen & Simmonds (2010)^9^,^51^ **	≥70°N	Oct–Jan	global
Wang *et al.* (2007)^52^	≥60°N	ann	global
Winton (2006, 2008)^12^,^39^	≥60°N	ann	global

We refer to the seasons used in defining Arctic amplification emphasized in the main body of the articles in question. ‘*all*’ corresponds to where results for all four seasons of Arctic amplification are clearly published in the article (note that in IPCC AR5 November-December is emphasized as a period of peak amplification but results are not produced anywhere explicitly in the main text).

^*^Difference between polar and tropical regions normalized by global temperature changes.

^**^Emphasizing rather the near surface air temperature change of the Arctic region (950–1000 hPa) compared with the entire tropospheric column (below around 300 hPa); in addition to Arctic amplification based on surface temperatures alone.
