# Supplementary material for: Problems encountered when defining Arctic amplification as a ratio
Source: Sci Rep. 2016 Jul 27;6:30469. doi: 10.1038/srep30469 (PMC4962034; doi:10.1038/srep30469)
Supplement: Supplementary Information [file srep30469-s1.pdf]

# 'Problems encountered when defining Arctic amplification as a ratio'

Supporting information

A. Hind, Q. Zhang and G Brattström

## Sensitivity and inconsistency in the Mean Ratio estimator, $r_{\mathrm{M}}$

As discussed in the main text, the Mean of Ratios ($r_{\mathrm{R}}$) and Ratio Mean ($r_{\mathrm{M}}$) are not identical ratio estimators:

$$\frac{E[a]}{E[g]} \approx E[r_{\mathrm{R}}] \neq E[r_{\mathrm{M}}] \tag{S1}$$

### Sensitivity when denominator ($\bar{g}$) not significantly different from 0

Here we demonstrate how the Mean Ratio estimator can be more sensitive to ensemble members with denominators not significantly different from 0 compared with the Ratio of Means estimator. A simple demonstration is conducted based on the model ensemble of Hwang *et al.*[1] (abbreviated as HW). This model ensemble consists of two different projected climate change forcing scenarios (A1B and A2) from the IPCC fourth assessment report with ten ($n$ = 10) simulations for each forcing experiment. In HW the Arctic amplification results are presented using the Mean Ratio estimator, where the two sets of ten simulations using either the A1B or A2 forcing scenarios have an Arctic amplification factor of **2.36** and **2.39** respectively (see their Table 1). Using a Ratio of Means estimator instead hardly changes the numbers at **2.38** and **2.41** respectively. Now consider a randomised weaker radiative forcing set of simulations with the same standard deviation for global and Arctic temperature change as in the A2 model ensemble, but with a smaller expected global temperature change of $E[g] = 0.5$°C and a smaller expected Arctic change of $E[a] = 1.2$°C. For reference, the sample mean of the global temperature change for the A2 model ensemble in HW is $\bar{g} = 2.34$°C and for the Arctic $\bar{a} = 5.56$°C, which become the expected population statistics in generating the randomised forcing data based on the A2 ensemble.

Figure S1 shows the Mean Ratio Arctic amplification factor as calculated from randomised data with increasing sample size (e.g. model ensemble size, $n$) drawn from population statistics based on the A2 forcing scenario ensemble of HW (red line) and the hypothetical weaker radiative forcing scenario (black line). Now contrast this with the Ratio of Means Arctic amplification factor in Fig. S2, which is for the exact same data as Fig. S1. Notice that in the data based on the A2 model spread of HW converges quickly near the value 2.4 for both the Mean Ratio (see embedded diagram in Fig. S1) and the Ratio of Means estimators beyond a small sample size ($n \sim$ 20-30). In the case of the weaker forcing scenario however inflated Arctic amplification factors based on the Ratio Mean estimator are possible in both negative and positive directions, even for very high sample sizes (Fig. S1). The Mean Ratio is clearly sensitive to individual ensemble members with denominators near 0, given that it attributes equal weighting to each ensemble members (see 'Ratio of Means or Mean Ratio?' in the main article).

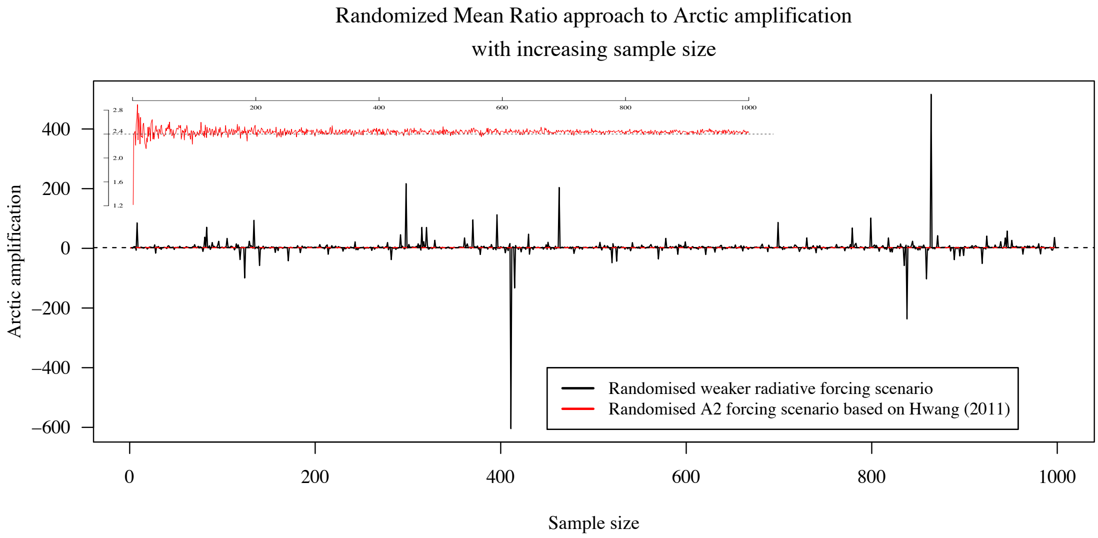


Figure S1: Mean Ratio estimate of Arctic amplification factor drawing with increasing sample size from a population distribution based on the A2 forcing scenario model ensemble of Hwang *et al.*[1] (red line) and then a weaker hypothetical forcing scenario population with the same standard deviation as the A2 case but with lower expected temperature change values for the global and Arctic (black line). The embedded red line is an enlargement of the randomized A2 scenario data.

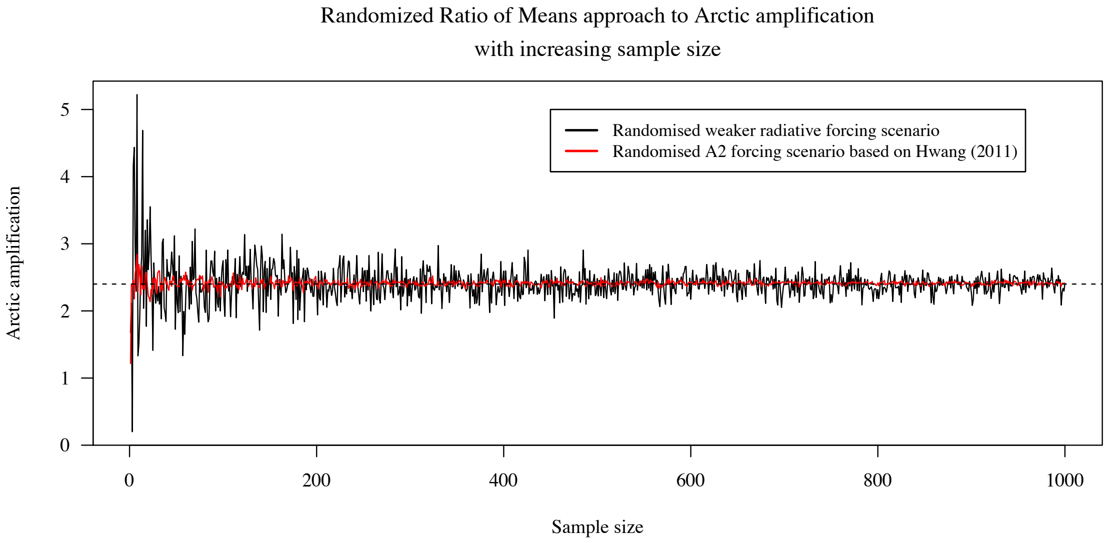


Figure S2: Same as Fig. S1 but for the Ratio of Means estimator of Arctic amplification.

The inflated values for the randomised hypothetical weaker forcing scenario data in Fig. S1, in practical terms could be avoided by simply removing any $r_i = a_i/g_i$ values from the ensemble that are exceptionally large due to $g_i$ values close to 0.

## Inconsistency

As discussed in the main text, through a second-order Taylor expansion of the ratio estimator $\frac{a}{g}$, Rao *et al.*[2] demonstrated that as sample size $n$ increases, the expected value of the Ratio of Means ($E[r_{\mathrm{R}}]$) approaches $\frac{E[a]}{E[g]}$, whereas the expected Mean Ratio statistic ($E[r_{\mathrm{M}}]$) does not, thus making the Mean Ratio estimator statistically inconsistent[3]. This behaviour is demonstrated through a randomised sampling that draws from the population distribution of the A1B forcing scenario ensemble in Hwang *et al.* in Fig. S3, for both the Ratio of Means (black line) and Mean Ratio (red line) statistics, against increasing sample size. The "true" Arctic amplification factor of the randomised data is in reality based on the sample statistics from the A1B forcing ensemble in HW, namely $\frac{E[a]}{E[g]} = \frac{5.56}{2.34} = 2.38$. In Figure S3, the Mean Ratio clearly does not approach the true amplification factor, supporting the conclusion of Rao[2] that the bias in the Mean Ratio estimator does not decrease with sample size and is hence statistically inconsistent.

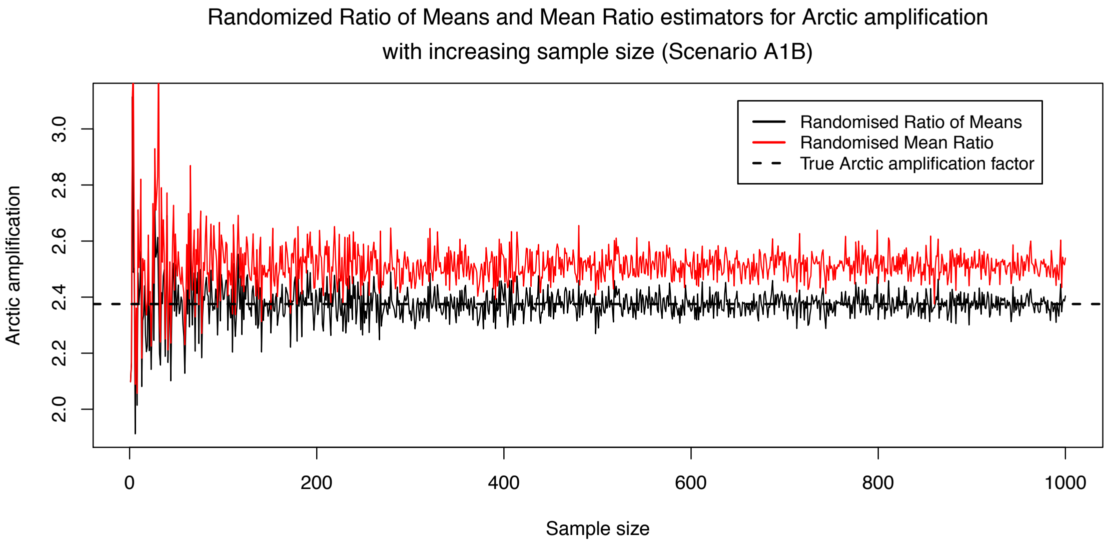


Figure S3: Ratio of Means (red) and Mean Ratio (black) estimators of the Arctic amplification factor, drawing with increasing sample size from a population distribution based on the A1B forcing scenario model ensemble of Hwang *et al.*[1]. The dashed line represents the true Arctic amplification value.

## References

1. Hwang, Y. T., Frierson, D. M. W. & Kay, J. E. Coupling between Arctic feedbacks and changes in poleward energy transport. *Geophys. Res. Lett.* **38,** L17704, (2011).

2. Rao, C. R. *Advanced Statistical Methods in Biometrical Research.* Wiley, New York, (1952).

3. Rao, T. J. Mean of ratios or ratio of means or both? *J. Stat. Plan. Infer.* **102,** 129–138, (2002).
